# Dielectric Function for Gold in Plasmonics Applications: Size Dependence of Plasmon Resonance Frequencies and Damping Rates for Nanospheres

**DOI:** 10.1007/s11468-015-0128-7

**Published:** 2015-11-14

**Authors:** Anastasiya Derkachova, Krystyna Kolwas, Iraida Demchenko

**Affiliations:** Institute of Physics, Polish Academy of Sciences, Al. Lotnikow 32/46, 02-668 Warsaw, Poland

**Keywords:** Gold, Gold nanoparticles, Dielectric function, Size effects, Localized surface plasmons (LSP), Mie theory, Dispersion relation, Plasmon resonance frequencies, Plasmon damping rates

## Abstract

Realistic representation of the frequency dependence of dielectric function of noble metals has a significant impact on the accuracy of description of their optical properties and farther applications in plasmonics, nanoscience, and nanotechnology. Drude-type models successfully used in describing material properties of silver, for gold are known to be not perfect above the threshold energy at 1.8 eV. We give the improved, simple dielectric function for gold which accounts for the frequency dependence of the interband transitions over 1.8 eV and, in addition, for the finite size effects in gold nanoparticles. On that basis, we provide the improved characterization of the spectral performance of gold nanoparticles. Furthermore, we give the direct size dependence of the resonance frequencies and total damping rates of localized surface plasmons of gold nanoparticles (retardation effects are taken into full account) in diverse dielectric environments. The results are compared to the data obtained experimentally for gold monodisperse colloidal nanospheres, as well with the experimental results of other authors.

## Introduction

Optical properties of matter are consequences of how it reflect, transmit, and absorb visible light. In many optical problems, the complex refractive index *n* of a material is the basic parameter. The index of refraction is related to the dielectric function (DF) *ε*(*ω*, **k**) which describes the electronic interaction of a medium with the incident light wave of frequency *ω* and wave vector **k.** In many problems, the general form of the dielectric function *ε*(*ω*, **k**) can be simplified to the spatially local function *ε*(*ω*, **k**) = *ε*(*ω*) = *n*(*ω*)^2^ = (*n*^′^(*ω*) + *in*^″^(*ω*))^2^ [1, 2]. In optics of metals, strong frequency dependence of *ε*(*ω*) is of basic importance in shaping their optical and transport properties. Significance of indexes of refraction noble metals in basic issues and applications has been a motivation to many experimental studies intended to increase the accuracy of measurements of their frequency dependence [3–7].

Optical properties of metal nanoparticles are known to be entirely different from their bulk counterparts. A major goal of nanoparticles’ science is to understand this intrinsic dissimilarity which manifests in observations and measurements. Despite dimensions smaller than the light wavelength, an electromagnetic (EM) wave is able to probe the details of nanoparticles structure. Basic optical properties of small particles can be explained satisfactorily by the classical EM theory using the bulk-type DF [8].

Noble metal nanoparticles attract great interest because of their outstanding optical properties which arise from their ability to resonate with light. Resonant excitation of localized surface plasmons (LSP) on nanoparticles give rise to a variety of effects, such as frequency-dependent absorption and scattering which can be tailored by particle dimensions [9–15]. Another advantage is the near-field concentration and enhancement which can be exploited for a variety of applications such as surface-enhanced Raman scattering (SERS), colorimetry, high-resolution microscopy, non-diffraction limited nanoscopic waveguides, or nanophotonic devices (see [16–21] for reviews). Resonance effects in nanoscale can be observed even with a necked eye and were empirically known and utilize since ancient times for coloring ceramics and glasses. LSP resonance (LSPR) frequencies depend strongly on nanoparticles shape, size, composition, and on the refractive index of immediate environment [11–14, 21–28]. Gradual understanding of the interaction of metallic nanostructures with light and explanation the physical processes which take place in such systems allows applying them as a variety of nanosensing modalities [21, 29–36], for photothermal cancer therapy [37], or in solar cells [38–41].

Currently, there are several numerical methods often used in predicting the scattering and absorption spectra of single nanoparticles. The set of Mie solutions to Maxwell’s equations (the Lorenz-Mie theory) is still the basic one. Originally [42], it described the scattering of EM plane wave by a homogeneous spherical particle. It is based on solutions of divergent-free Maxwell’s equations under the appropriate boundary conditions expressed in the form of an infinite series of spherical multipole partial waves. Another example of widely used approach for particles of any shape and limited dimensions is the finite-difference time-domain (FDTD) numerical technique [25, 43–45]. FDTD is an implementation of Maxwell’s time-dependent equations in partial differential form which are discretized by a grid mesh (Yee cells). The existence of scattering particle is defined by properly assigning the EM constants, including permittivity, permeability, and conductivity over the grid cells. However, the spectra of nanoparticles with various sizes which can be predicted using such methods provide only indirect information on how LSP properties change with size.

A more convenient direct method to describe LSP properties, such as resonance frequencies, spectral widths, radiative abilities, and number of modes involved, is to solve the dispersion relation for the surface localized EM fields [1, 11–15, 46]. Considering such LSP eigenmode problem in the absence of the incoming light field allowed to find the explicit size dependence of plasmon resonance frequencies and plasmon oscillation damping rates and delivers much more convenient and accurate tool for tailoring the plasmonic properties of nanoparticles [11–13, 21]. Solving such LSP eigenmode problem, the multipolar (e.g., dipole and higher order polarity) plasmon resonance frequencies and damping rates, with retardation effects taken into account, can be obtained as a smooth function of the particle radius for various indexes of refraction for the particle’s environment (e.g., [21]).

Realistic representation of the frequency dependence of DFs for metals has a significant impact on the results of electrodynamics calculations. Gold and silver nanostructures are most frequently used metals in either nanoscience or nanotechnology. They stand out due to high optical conductivity and chemical inertness under ambient conditions. Unfortunately, the models of dielectric function successfully used for silver (e.g., [2, 47]), for gold are known to be not perfect over the threshold energy of 1.8 eV. This motivated us to develop a better and simple analytical model of the DF for gold with the special emphasize on its applicability in plasmonics.

Consequently, the aim of this paper is to provide the improved analytic DF for gold with minimal number of parameters (“Dielectric Functions for Bulk Metals: Extended, Multi-Parameter Models” section) dedicated to plasmonic applications. Our modeling includes the previously unsolved problem of how to model the imaginary part of the DF in a simple analytic form in the frequency range over the absorption threshold energy at 1.8 eV. The proposed DF (“Analytically Simple Dielectric Function for Bulk Gold Accounting for Frequency-Dependent Interband Transitions” section) reproduces the corresponding experimentally measured real and imaginary parts of the index of refraction [4] in the energy range up to 3 eV. The proposed DF can be successfully used for bulk and nanostructured gold. On that basis, we study plasmonic properties of an exemplary nanostructure which is the gold nanosphere with size changing from single nanometers up to the large radii of hundreds nanometers. We give the direct description of the size dependence of LSP resonance frequencies and damping rates for divers indexes of refraction of dielectric environment (“Size Characterization of LSP Intrinsic Properties” section) and much improved modeling of spectral scattering and absorption abilities of gold nanoparticles (“Scattering and Absorption Spectra of Gold Nanospheres” section). In “Comparison of LSP Resonance Frequencies with the Experimental Results” section, these results are compared to the data which we obtained experimentally for gold colloids, as well with the experimental results of other authors [10, 48, 49].

**Table 1:** Some sets of the parameters *ε*_0_, *ω*_*p*_, *γ*_*bulk*_ of the dielectric functions *ε*_*Di*_(*ω*) (Eq. 2) reported in literature

	[5]	[11]	[44]	[51]	[52]	[53]	[54]
*ε*_0_	1	9.84	9.5	1.53	8.5	1	1
*γ*_*bulk*_ (eV)	0.026	0.072	0.06909	0.0729	0.0691	0.0184	0.07088
*ω*_*p*_ (eV)	9.02	9.01	8.9488	8.55	8.9517	8.55	8.89

## Models of Dielectric Function in Optical Issues

### Dielectric Function for Bulk Metals—Basic Models

Often used simple analytical form of the DF of metals results from the Drude-Sommerfeld model of perfect metal supplemented by electron relaxation after introducing the rate *γ*_*bulk*_:

1 $$ \varepsilon_{D}(\omega )=1-\frac{{\omega_{p}^{2}}}{\omega^{2}+i\gamma_{bulk}\omega }, $$

where *ω*_*p*_ is the bulk plasma frequency accounting for the number density of free electrons. The rate *γ*_*bulk*_ is proportional to the reciprocal of the mean free time between electron collisions in a metal. It can be determined from the electron mean free path *l* (42 nm for gold [9])as *γ*_*bulk*_∝*v*_*F*_/*l*, where *v*_*F*_ is the Fermi velocity. We express *ω*, *ω*_*p*_, and *γ*_*bulk*_ in electronvolts for convenience, as usual.

As known, noble metals are not perfect conductors at optical frequencies. More realistic but still simple Drude-like model with the effective parameters *ω*_*p*_ and *γ*_*bulk*_ accounts in addition the contribution of interband transitions to the polarizability by introducing of *ε*_0_ [11–14, 44, 50–52]:

2 $$\begin{array}{@{}rcl@{}} \varepsilon_{Di}(\omega )&=&\varepsilon_{0}-\frac{{\omega_{p}^{2}}}{ \omega^{2}+i\gamma_{bulk}\omega}\\ &=& \varepsilon_{0}-\frac{{\omega_{p}^{2}}}{\omega^{2}+\gamma_{bulk}^{2}}+i \frac{{\omega_{p}^{2}}\gamma_{bulk}}{\omega (\omega^{2}+\gamma_{bulk}^{2})} \end{array} $$

**Fig. 1:**
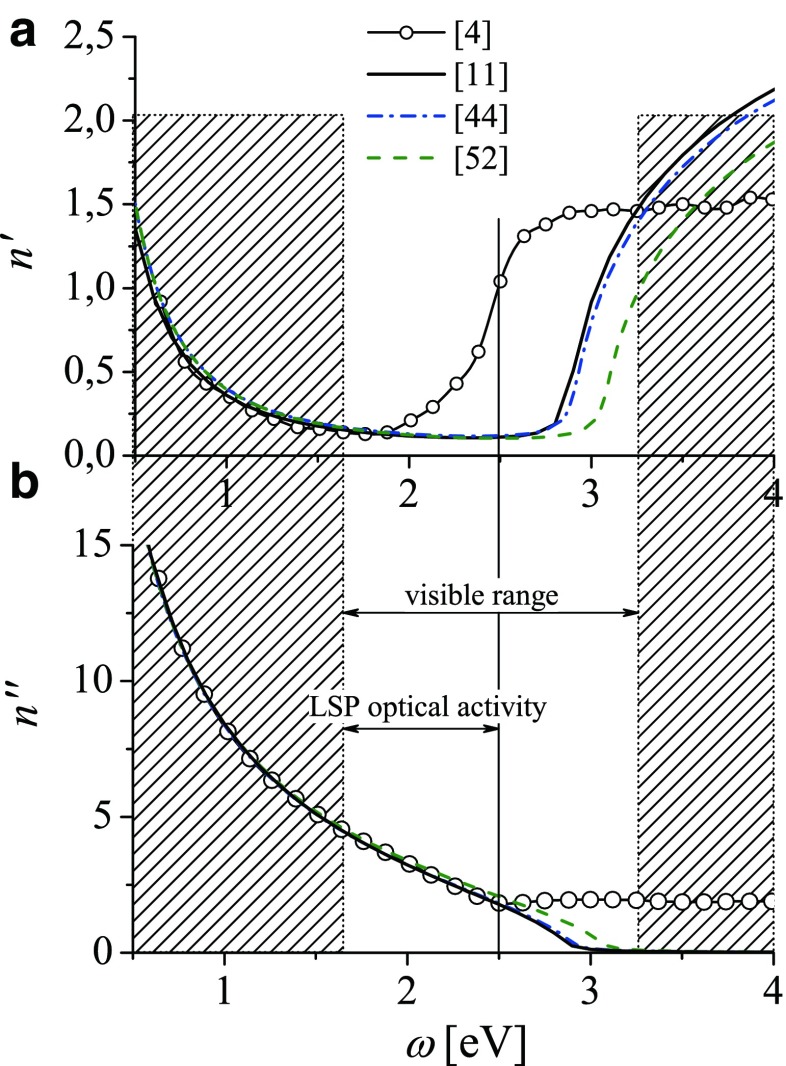
**a** Real and **b** imaginary parts of the refractive index for gold resulting from *ε*_*Di*_(*ω*) (2) with the parameters *ε*_0_, *ω*_*p*_, *γ*_*bulk*_ reported in [11, 44, 52] (*solid, dash-dot*, and *dashed lines* correspondingly) compared with the experimental data [4] (*line with circles*)

**Fig. 2:**
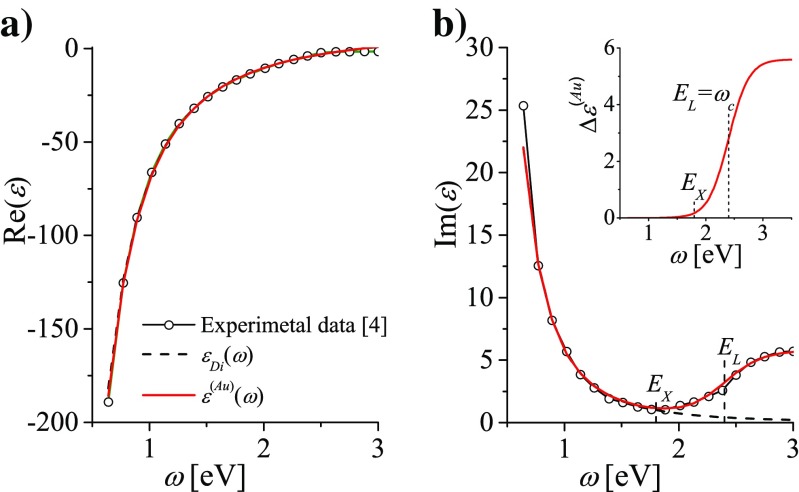
Comparison of the real (**a**) and (**b**) imaginary part of the dielectric function for gold resulting from different models. *Black dashed lines*: *ε*_*Di*_(*ω*) with parameters *ε*_0_ = 9.84, *ω*_*p*_ = 9.010eV, *γ* = 0.072eV. *Red solid lines*: *ε* ^(*Au*)^(*ω*) with the same parameters *ε*_0_, *ω*_*p*_ and *γ* but supplemented by *Δ* *ε* ^(*Au*)^(*ω*) (shown in the insertion) accounting for the frequency dependent interband transitions over 1.8 eV. Experimental data [4] are presented by the line with circles.

Such phenomenological model is used as a next step intended to better representing the frequency dependence of the experimental indexes of refraction for real metals such as gold and silver [3–7]. However, the reported effective parameters *ε*_0_, *γ*_*bulk*_, and *ω*_*p*_, which are usually claimed to result from the best fit [5, 44, 51–54] to the experimental data, are quite different (examples in Table 1 and Fig. 1). Moreover, the resulting real *n*^′^(*ω*) and imaginary *n*^″^(*ω*) parts of the refractive indexes for gold are not perfect in reproducing the experimental data (lines with circles) for larger photon energies of the optical range (Fig. 1). This is also the case of our previous studies of optical properties of gold nanoparticles [11–14] where the following effective parameters were accepted: *ε*_0_ = 9.84, *ω*_*p*_ = 9.010 eV, *γ*_*bulk*_ = 0.072 eV. Using of these parameters results in quite well fit (Fig. 2a, black line) of Re*ε*_*Di*_(*ω*) in the range up to 2.5 eV to the corresponding experimental values for gold [4] (line with circles). By turn, Im*ε*_*Di*_(*ω*) deviates strongly above the threshold energy at 1.8 eV, as shown in Fig. 2b for all the proposed sets of parameters. For silver in the optical range, such problem does not exist [47].

### Dielectric Functions for Bulk Metals: Extended, Multi-Parameter Models

There were several attempts [51, 55–57] to solve the problem of inaccuracy in modeling the optical properties of metals within an analytical model of DF in the visible/near-UV range. These are many-terms models including a large number of parameters, e.g., in [57] the multiparameter effective DF gives a good agreement with experimental data for gold after fitting four Lorentzian terms with 12 fitting parameters. Another example of a family of analytical models formulated in terms of so called critical points describing interband transitions in solids is reported in [51]. The proposed DF reproduces very well the experimental data [4] with eight fitting parameters. Such multi-term models are not algebraically simple, what in some issues can be not comfortable or make such models useless. The example can be the problem of dispersion relation for the surface localized plasmon fields [11–14]. In such issues, the frequency dependence of the DF convolves to the overall complex frequency dependence of the problem. If DF is too complicated, the problem is too difficult to be solved with the standard numerical methods.

With aim to simplify the DF for gold, we propose the improved intuitive Dude-like DF which would describe well the experimentally measured indexes of refraction [4] up to 3 eV after including frequency dependence of interband transitions in gold. This range (Fig. 1) contains the LSP optical activity of gold nanoparticles [10–14, 48, 49] (see Fig. 4 below).

### Frequency-Dependent Interband Transitions in Gold

In order to improve the phenomenological model of the dielectric function for optical and plasmonic applications based on the Drude-Sommerfeld model, let us shortly reconsider some data from the solid state physics. In particular, consideration of differences between gold and silver in that context seems to be very helpful.

It is known that metals exhibit characteristic shininess as their delocalized electrons are able to absorb and re-emit photons over a wide range of frequencies. Thus, the reflectance spectra of most metals are fairly flat and they appear silvery in color. Metallic properties of gold and silver atoms result from the valence electrons in the half-filled s-subshells. In Au, the electronic transition responsible for absorption is the transition from 5d to 6s level, in Ag it is 4d → 5s transition. However, in gold, relativistic effects raise energy of the 5d orbital and lower the 6s orbital [58] leading to the shift of absorption from ultraviolet to lower energies falling in the blue visible range. The relativistic effects in Ag are smaller than those in heavier Au: the 4d-5s distance in Ag is much greater; such transitions fall in the ultraviolet. As a result, the visible light is not absorbed but reflected equally: silver is silvery.

In bulk metals, the characteristic electronic band structure are formed with the state energy distribution resulting from the Pauli exclusion principle. The optical properties of metals depend on both intraband and interband transitions between electronic states [59, 60]. The strength of these transitions is determined by the energy dependent density of electronic states. In gold (near-) parabolic *sp*-hybridized conduction band, formed by lone *s*-electrons, is crossed by Fermi surface. Therefore, electrons in *sp*- band filled up to *E*_*F*_ = 5.53 eV, can move free (or rather quasi-free due to the electron scattering processes through collisions with metal ions). Interaction of light with such quasi-free conduction electrons is well described by *ε*_*D*_(*ω*) (1) for quasi-free electron gas ([9] and references therein).

Interband transitions are known to give an additive contribution to the dielectric function [9, 61] in some spectral frequency ranges. In gold, these transitions are closely related with electrons located in bands lying from 1 to 3 eV below the Fermi energy *E*_*F*_. Interband contributions depend on the location of the critical points, i.e., singularities, in the density of states which occur near symmetry points in the Brillouin zone. Near these points, so-called Van Hove singularities, the Fermi surface is deformed with respect to the spherical free electron surface. The large density of states in these regions is responsible for interband absorption and emission in the visible range. In gold, the interband transitions from the top of the *d* band to states just above *E*_*F*_ in the conduction band occur with the threshold at *E*_*L*_ = 2.4 eV in the visible range (below *λ* = 516.6 nm—blue light). Gold appears yellow because it absorbs blue light more than other colors of the visible spectrum. The reflected light is therefore lacking in blue compared to the incident white light what results in the yellowish tint, which is called the golden. The additional interband transition is due to the excitations of electrons from the 5*d*-band to unoccupied states in the 6*sp*-band above *E*_*F*_ with the interband gap *E*_*X*_ = 1.8 eV (light wavelength below *λ* = 688.8 nm—red light). The electromagnetic radiation of a wavelength in the vicinity of 600 nm is seen by a human as yellow.

### Analytically Simple Dielectric Function for Bulk Gold Accounting for Frequency-Dependent Interband Transitions

In the simplest models of the dielectric function, the interband transitions are taken into account (2) by introducing the constant *ε*_0_ instead of 1 for ideal free-electron metals. However, in gold, the strong frequency dependence of these transitions is not taken into account. It is the expected reason of why the applicability of $\text {Im} \varepsilon _{Di}(\omega )={\omega _{p}^{2}}\gamma _{bulk}/\omega (\omega ^{2}+\gamma _{bulk}^{2})$ (2) starts to deteriorate below 1.8 eV and breaks down over the second, more important threshold at about *E*_*L*_ = 2.4 eV. Consequently, our intuitive idea of accounting for these effects in the amended dielectric function *ε*^(*Au*)^(*ω*) is to modify *ε*_*Di*_(*ω*) by adding a simple, frequency dependent correction which could describe the interband transitions with the thresholds at *E*_*X*_ = 1.8eV and *E*_*L*_ = 2.4 eV. Inaccuracy in reproducing the experimental data by Im*ε*_*Di*_(*ω*) above 1.8eV (see Fig. 2) suggests, that such frequency dependent correction *Δε*^(*Au*)^(*ω*) should be added to Im*ε*_*Di*_(*ω*):

3 $$ \operatorname{Im}\varepsilon^{(Au)}(\omega )=\frac{\omega_{p}^{2} \gamma_{\text{bulk}}}{\omega (\omega^{2}+\gamma_{bulk}^{2})}+{\Delta}\varepsilon^{(Au)}(\omega ). $$

The frequency-dependent contribution of the interband transitions in gold we describe with a single, logistic function of two parameters: *A* and *Δ*:

4 $${\Delta} \varepsilon^{(Au)}(\omega)=\frac{A}{1+\exp (-(\omega -\omega_{c})/{\Delta})}.  $$

*Δε*^(*Au*)^(*ω*) starts at the lower asymptote at the zero level (see the inset in Fig. 2), and increases from *ω*≈1.6 eV, a little below the accepted lover energy gap of the interband transitions at *E*_*X*_ = 1.8 eV. It continues the fast increase crossing the central frequency *ω* = *ω*_*c*_≈*E*_*L*_ = 2.4 eV (the second energy gap of the interbad transitions). Farther it tends to the higher asymptote *A* = 5.6. Parameters *A* and *Δ* = 0.17 eV were chosen to reflect frequency dependence of the DF derived from the experimental data [4] in the range from 1eV to 3eV. We let Re*ε*_*Di*_(*ω*) unaffected:

5 $$ \text{Re}\varepsilon^{(Au)}(\omega )= \text{Re}\varepsilon_{Di}(\omega)= \varepsilon_{0}-\frac{{\omega_{p}^{2}}}{\omega^{2}+\gamma_{bulk}^{2}}. $$

So, the proposed form of *ε*^(*Au*)^(*ω*) for gold in optical and plasmonic applications, which includes the correction due to the frequency-dependent interband transitions is:

6 $$\varepsilon^{(Au)}(\omega )=\varepsilon_{Di}(\omega )+i{\Delta}\varepsilon^{(Au)}(\omega ). $$

with *Δε*^(*Au*)^(*ω*) approaching zero for *ω* below *E*_*X*_ = 1.8 eV. Figure 2 shows that *ε*^(*Au*)^(*ω*) (6) reproduces well the experimental data derived from [4] (see Fig. 2) in the studied frequency range and that the appropriateness of *ε*^(*Au*)^(*ω*) is much better than that of *ε*_*Di*_(*ω*). Noteworthy, in comparison with the models discussed in “Dielectric Functions for Bulk Metals: Extended, Multi-Parameter Models” section, applying *ε*^(*Au*)^(*ω*), we significantly reduced the number of terms and free parameters.

### Dielectric Function for Gold Nanoparticles Accounting for Frequency Dependent Interband Transitions and Finite Size Effects

Description of plasmons in nanosized noble metals through its local bulk DF fails dramatically when the particle size is smaller or comparable to the mean free path of conduction electrons [62]. In bulk metals, the collision time 1/*γ*_*bulk*_ is proportional to the electron mean free path, which at room temperatures for gold is equal to 42 nm [63]. When the mean free path becomes comparable or larger than a dimension of a particle, the effective collision time in such particles is greatly reduced. To account for this nonlocal effect near the metal interface, the additional phenomenological relaxation term *Cv*_*F*_/*R* is added to the relaxation rate *γ*_*bulk*_ [11–14, 62, 64–68]. *v*_*F*_ is the Fermi velocity (*v*_*F*_ = 1.4⋅10^6^ m/s), and *C* is the theory dependent quantity [64]. We accept the value *C* = 0.33 for gold nanoparticles, according to [69] and [66]. After including this correction to *ε*_*Di*_(*ω*), *ε*^(*Au*)^(*ω*) (6) is modified by the radius *R* if the nanosphere is sufficiently small:

7 $$\varepsilon^{(Au)}(\omega ,R)=\varepsilon_{Di}(\omega ,R)+i{\Delta}\varepsilon^{(Au)}(\omega ),  $$

where:

8 $$ \varepsilon_{Di}(\omega ,R)=\varepsilon_{0}-\frac{{\omega_{p}^{2}}}{\omega^{2}+i\left( \gamma_{bulk}+C\frac{v_{F}}{R}\right)\omega }. $$

*Δε*^(*Au*)^(*ω*) is given by Eq. 4. Let us note that surface scattering modifies strongly *ε*_*Di*_(*ω*, *R*) and so *ε*^(*Au*)^(*ω*, *R*) for relatively small radii only (see, e.g., [13]).

## Size Characterization of LSP Intrinsic Properties

### The Dispersion Relation for LSP Waves

The dispersion relation for LSP waves results from divergent free Maxwell equations [1, 11–14, 46] reduced to the Helmholtz homogeneous wave equations. Their vectorial solutions in two homogeneous regions inside and outside the sphere are expressed as a sum of infinite series of spherical multipole partial waves *l*, according to formalism of Mie scattering theory. However, the problem is formulated in absence of external, incoming light wave. The continuity relations at *r* = *R* for the tangential components of the transverse magnetic (TM) EM modes (with nonvanishing electric field component normal to the interface) lead to non-trivial solutions when:

9 $$\begin{array}{@{}rcl@{}} &&\sqrt{\varepsilon_{in}(\omega ,R)}\cdot \xi_{l}^{\prime }(k_{out}(\omega )\cdot R)\cdot\psi_{l}(k_{in}(\omega ,R)\cdot R)+ \\ &&-\sqrt{\varepsilon_{out}}\cdot \xi_{l}(k_{out}(\omega )\cdot R)\cdot \psi_{l}^{\prime }(k_{in}(\omega ,R)\cdot R)=0, \end{array} $$

where $k_{in}(\omega ,R)=\sqrt {\varepsilon _{in}(\omega ,R)}\cdot \omega /\hbar c$, and $k_{out}(\omega )=\sqrt {\varepsilon _{out}}\cdot \omega /\hbar c$ are the wave vectors inside the sphere, and in the sphere surroundings, respectively, *ε*_*in*_(*ω*, *R*) and *ε*_*out*_ are DFs of the metal sphere and of the dielectric environment, respectively. The complex *ψ*_*l*_(*z*), *ξ*_*l*_(*z*) are Riccati-Bessel spherical functions (of complex arguments) which can be expressed by the Bessel *J*_*l*+1/2_(*z*), Hankel $H_{l+1/2}^{(1)}(z),$ and Neuman *N*_*l*+1/2_(*z*) cylindrical functions of the half order, and *c* is the speed of light. The corresponding equations for the transverse electric (TE) mode has no solution for Re*ε*_*in*_(*ω*, *R*)<0, as in the local case [1].

Solutions of the dispersion relation (9) depend strongly on the form of DFs for the particle and its surroundings and exist only for the complex frequencies $\omega _{l}^{\prime }(R)+i\omega _{l}^{\prime \prime }(R)$ of the surface TM modes *l* on a sphere of radius *R* [11–14, 46, 47]. The oscillation frequencies $\omega _{l}^{\prime }(R) $ of the surface localized fields (plasmon modes) and the damping rates $|\omega _{l}^{\prime \prime }(R)| $ of these oscillations ($\omega _{l}^{\prime \prime }(R)<0$) can be found numerically for known DFs *ε*_*in*_(*ω*, *R*) and *ε*_*out*_ for successive *R*. Let us stress, that the form of the function *ε*_*in*_(*ω*, *R*) strongly influences the resulting $\omega _{l}^{\prime }(R)$ and $\omega _{l}^{\prime \prime }(R)$ dependencies due to specific interplay of frequency dependence of all involved functions; *ε*_*in*_(*ω*, *R*) convolves to the overall dependence of the complex *ψ*_*l*_(*z*), *ξ*_*l*_(*z*) functions of frequency dependent (complex) arguments. In fact, *ε*_*in*_(*ω*, *R*) in the dispersion relation (9) should be in the analytic form. It can not be replaced by its numerical values when looking for roots of Eq. 9. Proper $\omega _{l}^{\prime }(R)$ and $\omega _{l}^{\prime \prime }(R)$ dependencies can be found only with the realistic model of *ε*_*in*_(*ω*, *R*).

### LSP Resonance Frequencies and Damping Rates as a Function of Radius

Let us stress that not only LSP resonance frequencies but also both $\omega _{l}^{\prime }(R)$ and $\omega _{l}^{\prime \prime }(R)$ are necessary to understand and control the spectral performance of nanoparticles and the manner it changes with the radius *R*. The damping of plasmon mode oscillations (described by $|\omega _{l}^{\prime \prime }(R)|$) consist of the radiative and dissipative damping processes with the size dependent contributions. Plasmon modes characterized by small radiative damping are optically inactive. Increasing contribution of the radiative damping to the total plasmon damping leads to suppression of the dissipative channel by the increasing radiative processes for larger *R* and *l* [13]. The plasmon resonance takes place when the optically active plasmon mode *l* (*l* = 1,2,3...) is excited by EM field of frequency $\omega =\omega _{l}^{\prime }(R)$. Excited plasmon oscillations are damped with the corresponding damping rates $|\omega _{l}^{\prime \prime }(R)|$.

**Fig. 3:**
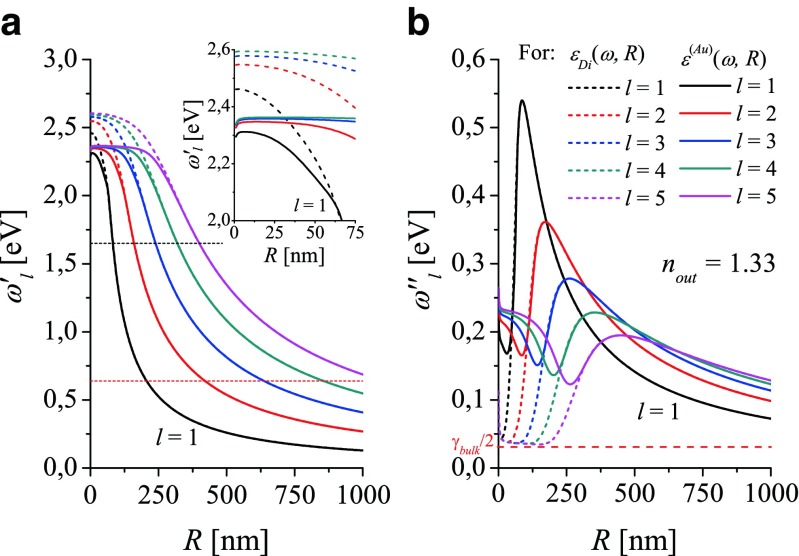
Comparison of (**a**) LSP resonance frequencies $\omega _{l}^{\prime }(R)$ and (**b**) damping rates $\omega _{l}^{\prime \prime }(R)$ calculated vs radius for different models of the dielectric function; *dashed lines*: for *ε*_*Di*_(*ω*, *R*); *solid lines*: for *ε* ^(*Au*)^(*ω*, *R*) accounting for contribution of the frequency-dependent interband transitions over 1.8eV. *n*_*out*_ = 1.33 (water). *Horizontal short-dashed lines* (**a**) show the lower frequency limits: of the optical range (*red line* at 1.65 eV) and of the measured indexes of refraction [4] (*black line* at 0.64 eV (see Fig. 1)

Figure 3 illustrate the $\omega _{l}^{\prime }(R)$ and $|\omega _{l}^{\prime \prime }(R)|$ dependencies calculated with the dielectric function *ε*_*in*_(*ω*, *R*) resulting from different models (see Eqs. 2 and 7): *ε*_*in*_(*ω*, *R*) = *ε*_*Di*_(*ω*, *R*)—dashed lines, and *ε*_*in*_(*ω*, *R*) = *ε*^(*Au*)^(*ω*, *R*) - solid lines, for gold nanospheres with *R* changing from 1nm to 1000 nm for modes *l* starting from the dipole mode with *l* = 1 up to *l* = 5. Here, $n_{out}=\sqrt {\varepsilon _{out}}=$1.33 (water). Black lines (solid and dashed correspondingly) represent the dipole resonance frequencies $\omega _{l=1}^{\prime }$ resulting from both models of DF.

Comparison of $\omega _{l}^{\prime }(R)$ and $|\omega _{l}^{\prime \prime }(R)|$ resulting from the standard *ε*_*Di*_(*ω*, *R*) and the new *ε*^(*Au*)^(*ω*, *R*) DFs (see Fig. 3a) reveals strong impact of the model of DF applied to EM calculations, as expected. Application of *ε*^(*Au*)^(*ω*, *R*) results in an important modification of resonance frequencies $\omega _{l}^{\prime }(R)$ which are shifted by ∼0.15 eV towards smaller frequencies. In the optical range, their size dependence vs *R* is weaker (less than 30 % for the dipole mode). Such EM modeling reveals also the red shift of the LSP resonance frequencies with the decreasing *R* in the smallest particle range (see the inset in Fig. 3a) in addition to the red shift with increasing *R* (due to EM retardation) for larger size ranges.

The total damping rates $|\omega _{l}^{\prime \prime }(R)|$ of plasmon modes *l* are even more strongly affected (see Fig. 3b) by the model of DF (and the effects included in that modelling). To understand what are the underlying phenomena, let us notice that in the case of simplest model of DF (*ε*_*D*_(*ω*), Eq. 2), $|\omega _{l}^{\prime \prime }(R)|$ start to increase from the value *γ*_*bulk*_/2 ([13]) which is the nonradiative plasmon damping rate common for all modes in the smallest particle range (Fig. 3b dashed lines). After applying *ε*_*Di*_(*ω*) (2) in calculations, $|\omega _{l}^{\prime \prime }(R)|$ are modified by the finite size effect (see “Dielectric Function for Gold Nanoparticles Accounting for Frequency Dependent Interband Transitions and Finite Size Effects” section) in the smallest nanosphere range, as presented in Fig. 3b (dashed lines). In that range, $|\omega _{l}^{\prime \prime }(R)|$ decrease with increasing *R*. Farther strong increase of $|\omega _{l}^{\prime \prime }(R)|$ is due to the increasing radiative damping. $2|\omega _{l}^{\prime \prime }|$ defines the spectral widths of maxima in the corresponding spectra (see, e.g., [10, 12, 13]). However, existence of such narrow plasmon resonances as those resulting from applying *ε*_*D*_(*ω*) or *ε*_*Di*_(*ω*) is not confirmed experimentally. Fortunately, application of the realistic DF for gold (7) solves this problem. As shown in Fig. 3b (solid lines), application of *ε*^(*Au*)^(*ω*, *R*) augments $|\omega _{l}^{\prime \prime }(R)|$ by the order of magnitude giving the realistic spectral widths for *l* = 1 (the experimental data exist only for the dipole mode [10]).

Both the plasmon resonance oscillation frequencies $\omega _{l}^{\prime }(R)$ and damping rates $|\omega _{l}^{\prime \prime }(R)|$ characterize the intrinsic plasmonic properties of gold nanospheres of any size. These properties are reflected in the spectra of light scattered or absorbed by such particle derived from Mie scattering theory.

**Fig. 4:**
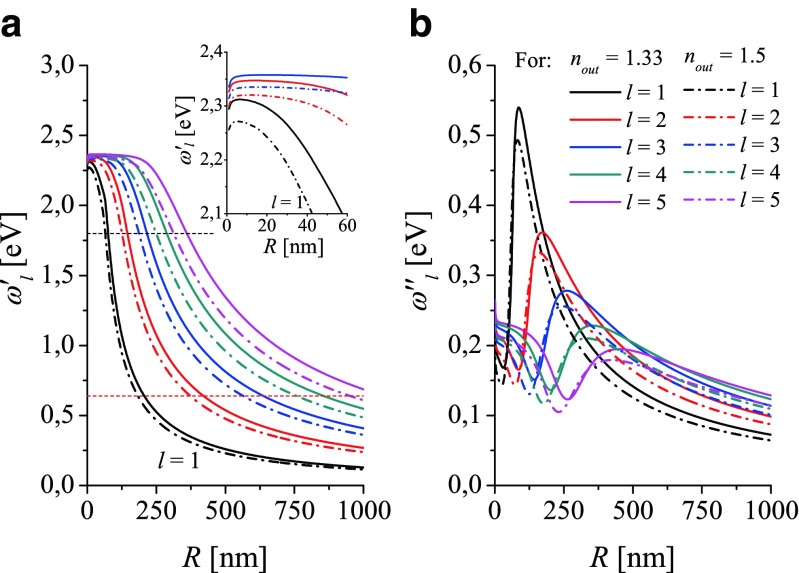
Comparison of (**a**) LSP resonance frequencies $\omega _{l}^{\prime }(R)$ and (**b**) damping rates $\omega _{l}^{\prime \prime }(R)$ calculated vs radius for gold nanospheres immersed in water (*n*_*out*_ = 1.33, *solid lines*) and in oil (*n*_*out*_ = 1.5, *dot-dashed lines*). Calculations are performed using *ε* ^(*Au*)^(*ω*, *R*) (7). *Horizontal short-dashed lines* (**a**) show the lower frequency limit: of the optical range (*red line* at 1.65 eV) and of the measured indexes of refraction [4] (*black line* at 0.64 eV (see Fig. 1))

The plasmon resonance frequencies $\omega _{l}^{\prime }(R)$ and damping rates $|\omega _{l}^{\prime \prime }(R)|$ are very sensitive to refractive index of surrounding medium ($n_{out}=\sqrt {\varepsilon _{out}}$), what can be used in many applications (see, e.g., [21]). Figure 4 demonstrates this effect for *n*_*out*_ = 1.33 (water) and *n*_*out*_ = 1.5 (immerse oil). The resonance frequencies $ \omega _{l}^{\prime }(R)$ (Fig. 4a) undergo red shift with increasing *n*_*out*_. The corresponding damping rates $|\omega _{l}^{\prime \prime }(R)|$ are shifted toward higher or lower value, depending on the size range which change with *l*, as demonstrated in Fig. 4b.

## Scattering and Absorption Spectra of Gold Nanospheres

One of the most frequently used quantities for describing the spectral properties of spherical particles illuminated by a plane wave are the corresponding cross-sections calculated from Mie theory [8, 42, 70]. The extinction *C*_*ext*_, absorption *C*_*abs*_, and scattering *C*_*scat*_ cross-sections are expressed by series expansion of the involved fields into partial waves:

10 $$ C_{scat}=\frac{2\pi }{k_{out}^{2}}\sum\limits_{l=1}^{\infty }(2l+1)(|a_{l}|^{2}+|b_{l}|^{2}), $$

11 $$ C_{ext}=\frac{2\pi }{k_{out}^{2}}\sum\limits_{l=1}^{\infty }(2l+1)\text{Re} \left( {a_{l}^{2}}+{b_{l}^{2}}\right), $$

12 $$ C_{abs}=C_{ext}-C_{scat}, $$

with the coefficients *a*_*l*_ and *b*_*l*_:

13a $$\begin{array}{@{}rcl@{}} a_{l}&=&\frac{m\psi_{l}(mx)\psi_{l}^{\prime }(x)-\psi_{l}(x)\psi_{l}^{\prime }(mx)}{m\psi_{l}(mx)\xi_{l}^{\prime}(x)-\xi_{l}(x)\psi_{l}^{\prime }(mx)}, \end{array} $$

13b $$\begin{array}{@{}rcl@{}} b_{l} &=& \frac{\psi_{l}(mx)\psi_{l}^{\prime}(x)-m\psi_{l}(x)\psi_{l}^{\prime }(mx)}{\psi_{l}(mx)\xi_{l}^{\prime }(x)-m\xi_{l}(x)\psi_{l}^{\prime }(mx)}. \end{array} $$

**Fig. 5:**
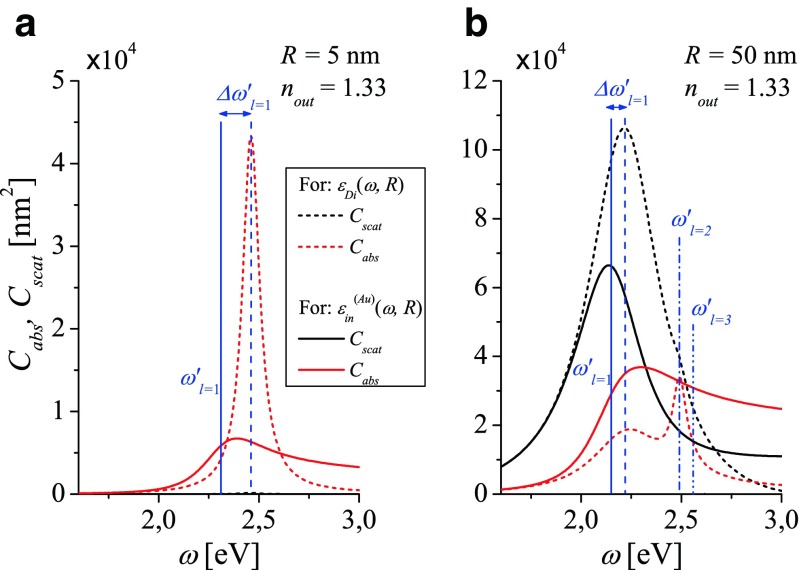
Comparison of the absorption *C*_*abs*_ (*red lines*) and scattering *C*_*scat*_ (*black lines*) cross sections calculated for different models of the dielectric function; *dashed lines*: for *ε*_*Di*_(*ω*, *R*); *solid lines*: for *ε* ^(*Au*)^(*ω*, *R*) for gold nanospheres with the radius (**a**) *R* = 5 nm and (**b**) *R* = 50 nm embedded in water. *Vertical solid and dashed lines* show correspondingly the position of LSP resonance frequencies $\omega _{l}^{\prime }(R)$ resulting from the dispersion relation (9) for both models of the dielectric function

*x* = 2*πR*/*λ* is the size parameter, *λ* is the wavelength of the incident light wave in vacuum, *m* = *n*_*in*_/*n*_*out*_. Spectral properties of metal nanosphere described by *C*_*ext*_(*ω*), *C*_*abs*_(*ω*) and *C*_*scat*_(*ω*) are dominated by plasmon resonances (often expected to point the intrinsic value of LSPR at $\omega _{l}^{\prime }(R)$) which manifest in the corresponding spectra in different manner. The spectral position of the maxima, their number, heights, and bandwidths change with *R*. Mie predictions concerning the spectral characteristics of gold nanoparticles are dramatically dependent on the model of its DF (see Fig. 5). The absorption (red lines) and scattering spectra (black lines) calculated with *ε*^(*Au*)^(*ω*, *R*) (solid lines) and *ε*_*Di*_(*ω*, *R*) (dashed lines) for gold nanospheres of radius *R* = 5 nm (Fig. 5a) and *R* = 50 nm (Fig. 5b) are presented for comparison.

In case of a small nanosphere (*R* = 5 nm), absorption dominates over scattering, as illustrated in Fig. 5a: *C*_*scat*_(*ω*) is negligible in comparison with *C*_*abs*_(*ω*) in the whole optical range. *C*_*abs*_(*ω*) calculated with *ε*^(*Au*)^(*ω*, *R*) (solid red line) becomes spectrally broader and is shifted towards smaller *ω* compared to the predictions using *ε*_*Di*_(*ω*, *R*) (dashed red line) in qualitative agreement with the predicted shift $\varDelta \omega _{l=1}^{\prime } $ of the dipole plasmon resonance frequencies $\omega _{l=1}^{\prime }(R)$ (see Fig. 3). However, the position of the peak in *C*_*abs*_(*ω*) calculated with *ε*^(*Au*)^(*ω*, *R*) does not coincide with the dipole plasmon resonance frequency $\omega _{l=1}^{\prime } $ (vertical solid line) obtained from the dispersion relation (9) for the surface localized fields. In case of applying *ε*_*Di*_(*ω*, *R*) in calculations, position of the peak in *C*_*abs*_(*ω*) (shown with the red dashed line) coincides with $\omega _{l=1}^{\prime } $ (vertical dashed line) in small (Fig. 5a) and large (Fig. 5b) nanospheres. The shift $\varDelta \omega _{l}^{\prime } $ is due to the fact, that we included the frequency-dependent interband transitions in the model of *ε*^(*Au*)^.

Absorption of light by larger gold sphere with radius *R* = 50 nm, (Fig. 5b, solid red line) is less efficient than scattering (solid black line). Absorption and scattering peaks are spectrally shifted in respect to each other. The scattering peak coincides with $\omega _{l=1}^{\prime }(R) $ resulting from the dispersion relation (9) for the surface localized fields. Both absorption and scattering spectra calculated with *ε*^(*Au*)^(*ω*, *R*) (solid lines) are qualitatively and quantitatively different if compared with those calculated for *ε*_*Di*_(*ω*, *R*) (dashed lines).

One can conclude that in gold nanoparticles (after accepting *ε*^(*Au*)^(*ω*, *R*) (7), the peak in computed scattering spectra better reflect the spectral position of the dipole LSP resonances than the peak in absorption spectra. For silver nanoparticles well described by *ε*_*Di*_(*ω*, *R*), it is opposite: the peaks in absorption spectra better reproduce the spectral manifestation of LSP resonances.

**Fig. 6:**
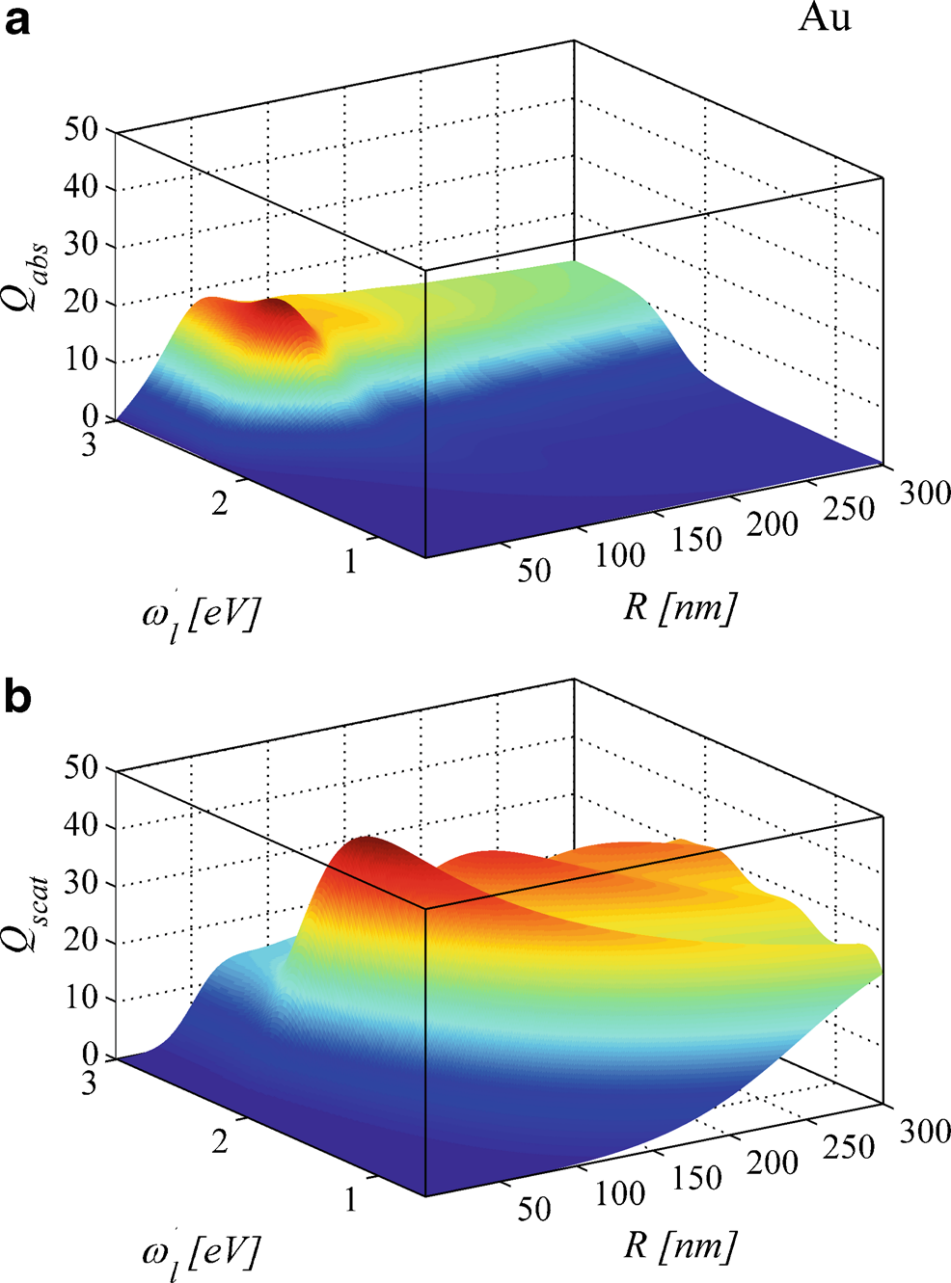
3D plots illustrating the efficiency of **a** absorption *Q*_*abs*_(*ω*, *R*) and **b** scattering *Q*_*scat*_(*ω*, *R*) of gold nanospheres as predicted by Mie theory with *ε* ^(*Au*)^(*ω*, *R*) and *n*_*out*_ = 1

3D maps in Fig. 6 summarize spectral performance of gold nanoparticles as a function of *R*. It illustrates spectral efficiencies *Q*_*abs*_(*ω*, *R*) = *C*_*abs*_(*ω*, *R*)/*πR*^2^ and *Q*_*scat*_(*ω*, *R*) = *C*_*scat*_(*ω*, *R*)/*πR*^2^ of gold nanoparticles in the frequency range from 0.64 eV up to 3 eV, where *ε*^(*Au*)^(*ω*, *R*) have been proven to realistically describe the interaction of light with gold nanoparticles. The input parameters are *ε*^(*Au*)^(*ω*, *R*) (7) and *n*_*out*_ = 1. In particular, Fig. 6 shows that the maximal efficiency of absorption falls in different spectral and size ranges than the maximal efficiency of scattering.

## Comparison of LSP Resonance Frequencies with the Experimental Results

**Fig. 7:**
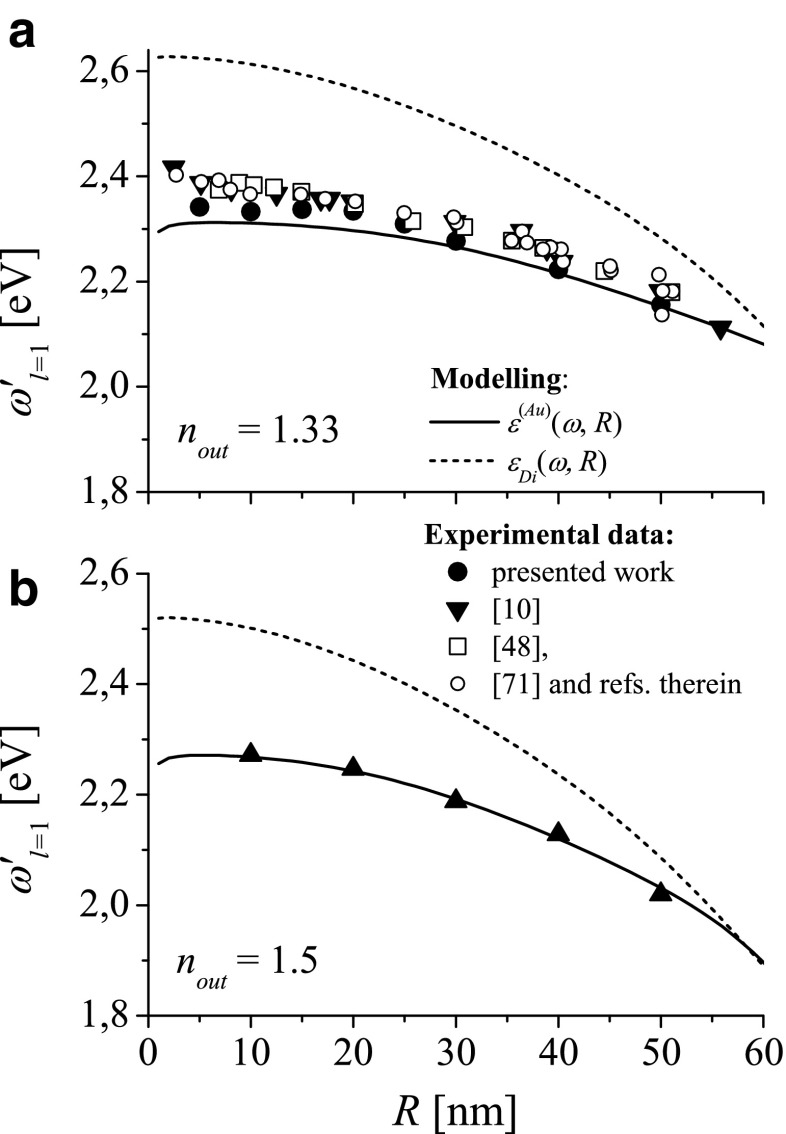
Comparison of the dipole resonance frequencies derived from the experimental data [10, 48, 71] and from the present work (*closed circles*) for gold nanosphere embedded (**a**) in water (*n*_*out*_ = 1.33) and (**b**) in immerse oil (*n*_*out*_ = 1.5) with $\omega _{l=1}^{\prime }(R)$ calculated for different models of the dielectric function; *dashed lines*: for *ε*_*Di*_(*ω*, *R*); *solid lines*: for *ε* ^(*Au*)^(*ω*, *R*)

Figure 7 shows the comparison of experimental data with predictions for $\omega _{l=1}^{\prime }(R)$ (solid and dashed lines) derived from dispersion relation (9). Solid line shows $\omega _{l=1}^{\prime }(R)$ calculated for *ε*_*in*_(*ω*, *R*) = *ε*^(*Au*)^(*ω*, *R*) (7) and dashed line—for *ε*_*Di*_(*ω*, *R*) (8) respectively.

Experimental data in Fig. 7 show spectral positions of peaks determined from the experimental spectra of mono-disperse or single gold nanospheres as a function of radius *R* embedded in water (Fig. 7a) or in immerse oil (Fig. 7b). Peak positions determined from the absorption spectra comes from [48] (open squares), [71] (open circles and triangles), this work (closed spheres), and those from the scattering spectra of single nanoparticles comes from [10] (closed triangles).

Our experimental results marked with closed circles in Fig. 7a are taken for commercial unconjugated gold colloids, produced by BBI (British BioCell International) supplied in water with concentration from 5.6⋅10^9^ particles/ml (for 100 nm spheres) to 5.7⋅10^12^ particles/ml (for 10 nm spheres). The particles are citrate stabilized with a net negative surface charge. Absorption spectra of gold colloid were gathered in transmission detection mode using USB 2.0 Fiber Optic Spectrometer (USB 4000 Ocean Optics, B. V.). The source light was focused by fiber optic taper (FOCON).

Figure 7 shows that the peaks (ascribed to dipole resonance) obtained from the experimental absorption and scattering spectra are quite similar: their difference is not larger than the experimental and model-dependent errors, when using *ε*_*Di*_(*ω*, *R*) for deconvolving the peak positions from the experimental spectra. As demonstrated in Fig. 5b (dashed black and red line), the absorption and scattering peaks lye very nearby. Let us note that the spectral peak positions of the experimental spectra are very sensitive to impurities in the composition of both the nanospheres and environment. Minor modification in the composition of nanosphere/environment material affects the indexes of refraction (DFs) and results in red/blue shift of LSP peak position. To reduce these effect, nanospheres serving for accurate LSP size characterization are usually chemically stabilized and possess a thin coat with the refractive index other then nanosphere material. This fact is an additional reason in a small discrepancy between the LSPR frequencies derived from the experimental spectra and the numerically predicted $\omega _{l=1}^{\prime }(R)$ (Figs. 4 and 7).

Figure 7 shows also that $\omega _{l=1}^{\prime }(R)$ calculated for *ε*_*in*_(*ω*, *R*) = *ε*^(*Au*)^(*ω*, *R*) describes much better size dependence of the experimental data than $\omega _{l=1}^{\prime }(R)$ calculated for *ε*_*Di*_(*ω*, *R*).

## Conclusions

The existing analytical models of the dielectric function successfully used in describing plasmonic properties of silver, for gold are known to be not perfect over the threshold energy of 1.8 eV, especially in its imaginary part. The reason is that analytical simple models of the dielectric function, which are often used in practice, account for the interband transition contribution to the polarizability by a constant which improves the Drude model of perfect metals. However, in gold, the interband transitions occur with the thresholds in visible range and display strong frequency dependence. This is the reason why the applicability of $\text {Im}\varepsilon _{Di}(\omega )={\omega _{p}^{2}}\gamma _{bulk}/\omega (\omega ^{2}+\gamma _{bulk}^{2})$ (Eq. (2)) breaks down starting from about 1.8 eV and collapses over 2.4 eV. We give the improved, but still simple analytic Dude-like DF which describes well the experimental data of [4] in the energy range up to 3 eV. This is the range of plasmonic activity of gold nanoparticles [10–14, 48, 49] of sizes from single nanometers up to the radius of hundreds of nanometers. The derived dielectric functions *ε*^(*Au*)^(*ω*) (3) for bulk gold is adapted for gold nanospheres *ε*^(*Au*)^(*ω*, *R*) (7) by taking into account the finite size effect. Such functions used in electrodynamic calculations allow more accurate prediction of many optical phenomena involving bulk and nanoscaled gold.

In particular, we found realistic multipolar plasmon resonance frequencies and plasmon damping rates for gold spheres by solving the dispersion relation for surface localized EM waves and compared these predictions with the data extracted from the experimental spectra measured for gold colloidal monodisperse nanospheres. We also included the experimental results of other authors [10, 48, 71] and proved much better applicability of our electrodynamic modelling with the derived dielectric function in the description of absorption, extinction, and scattering spectra of gold nanospheres with various radii. In particular, our data describe much better the size dependence of multipolar LSP resonance frequencies and total dumping rates.

## References

[1] Fuchs R, Halevi P (1992) Basic Conce pts and Formalism of Spatial Dispertion, in Spatial Dispertion in Solids and Plasmas. North-Holland

[2] Maier SA (2007) Plasmonics: Fundamentals and Applications. Springer ScienceBusinessMedia LLC

[3] Guerrisi M, Rosei R (1975). Splitting of the interband absorption edge in An. Phys Rev B.

[4] Johnson P, Christy R (1972). Optical constants of noble metals. Phys Rev B.

[5] Ordal MA, Bell RJ, Alexander RW, Long LL, Querry MR (1985). Optical properties of fourteen metals in the infrared and far infrared: Al, Co, Cu, Au, Fe, Pb, Mo, Ni, Pd, Pt, Ag, Ti, V, and W. Appl Opt.

[6] Palik ED (1998). Handbook of Optical Constants of Solids.

[7] Blanchard NP, Smith C, Martin DS, Hayton DJ, Jenkins TE, Weightman P (2003). High-resolution measurements of the bulk dielectric constants of single crystal gold with application to reflection anisotropy spectroscopy. Phys Stat Sol C.

[8] Bohren CF, Huffman DR (1983) Absorption and scattering of light by small particles. Wiley science paperback series, Wiley

[9] Kreibig U, Vollmer M (1995). Optical Properties of Metal Clusters.

[10] Sönnichen C, Franzl T, Wilk T, von Plessen G, Fe J (2002). Plasmon resonances in large noble-metal clusters. New J Phys.

[11] Derkachova A, Kolwas K (2007). Size dependence of multipolar plasmon resonance frequencies and damping rates in simple metal spherical nanoparticles. Eur Phys J-Spec Top.

[12] Kolwas K, Derkachova A, Shopa M (2009). Size characteristics of surface plasmons and their manifestation in scattering properties of metal particles. JQSRT.

[13] Kolwas K, Derkachova A (2013). Damping rates of surface plasmons for particles of size from nano- to micrometers; reduction of the nonradiative decay. JQSRT.

[14] Derkachova A, Kolwas K (2013). Simple analytic tool for spectral control of dipole plasmon resonance frequency for gold and silver nanoparticles. Photonics Letters of Poland.

[15] Bakhti S, Destouches N, Tishchenko AV (2015) Singular representation of plasmon resonance modes to optimize the near- and far-field properties of metal nanoparticles. Plasmonics:1–9

[16] Wang Y, Plummer E, Kempa K (2011). Foundations of Plasmonics. Adv Phys.

[17] Quinten M (2011) Optical Properties of Nanoparticle Systems. Wiley-VCH Verlag GmbH &amp; Co. KGaA

[18] Aslan K, Lakowicz JR, Geddes CD (2005). Plasmon light scattering in biology and medicine: new sensing approaches, visions and perspectives. Curr Opin Chem Biol.

[19] Willets K, Van Duyne R (2007). Localized surface plasmon resonance spectroscopy and sensing. Annu Rev Phys Chem.

[20] Brongersma M, Hartman J, Atwater H (2000). Electromagnetic energy transfer and switching in nanoparticle chain arrays below the diffraction limit. Phys Rev B.

[21] Kolwas K, Derkachova A, Jakubczyk D (in print) Advances in Nanomaterials and Tissue Engineering. Apple Academic Press

[22] Jain K, Eustis S, El-Sayed M (2006). Optical properties of metal clusters (springer series in material science). J Phys Chem B.

[23] Bukasov R, Shumaker-Parry J (2007). Highly tunable infrared extinction properties of gold nanocrescents. Nano Lett.

[24] Zhu J (2007). Ellipsoidal core-shell dielectric-gold nanostructure: theoretical study of the tunable surface plasmon resonance. J Nanosci Nanotechnol.

[25] Dmitriev A, Pakizeh T, Kall M, Sutherland D (2007). Gold-silica-gold nanosandwiches: tunable bimodal plasmonic resonators. Small.

[26] Halas N (2005). Playing with plasmons: tuning the optical resonant properties of metallic nanoshells. MRS Bull.

[27] Stockman M, Li K, Brasselet S, Zyss J (2006). Octupolar metal nanoparticles as optically driven, coherently controlled nanorotors. Chem Phys Lett.

[28] Zhang JZ, Noguez C (2008). Plasmonic optical properties and applications of metal nanostructures. Plasmonics.

[29] Henglein A (1993). Physicochemical properties of small metal particles in solution: “microelectrode” reactions, chemisorption, composite metal particles, and the atom-to-metal transition. J Phys Chem.

[30] Jackson J, Westcott S, Hirsch L, West J, Halas N (2003). Controlling the surface enhanced raman effect via the nanoshell geometry. Appl Phys Lett.

[31] Linnert T, Mulvaney P, Henglein A (1993). Surface chemistry of colloidal silver: surface plasmon damping by chemisorbed iodide, hydrosulfide (sh-), and phenylthiolate. J Phys Chem.

[32] Mulvaney P (1996). Surface plasmon spectroscopy of nanosized metal particles. Langmuir.

[33] Nath N, Chilkoti A (2002). A colorimetric gold nanoparticle sensor to interrogate biomolecular interactions in real time on a surface. Anal Chem.

[34] Okamoto T, Yamaguchi I, Kobayashi T (2000). Local plasmon sensor with gold colloid monolayers deposited upon glass substrates. Opt Lett.

[35] Tam F, Halas N (2003). Plasmon response of nanoshell dopants in organic films: a simulation study. Prog Org Coat.

[36] Tam F, Moran C, Halas N (2004). Geometrical parameters controlling sensitivity of nanoshell plasmon resonances to changes in dielectric environment. J Phys Chem B.

[37] O’Neal D, Hirsch L, Halas N, Payne J, West J (2004). Photo-thermal tumor ablation in mice using near infrared-absorbing nanoparticles. Cancer Lett.

[38] Catchpole KR, Polman A (2008) Design principles for particle plasmon enhanced solar cells. Appl Phys Lett 93(191113)

[39] Catchpole KR, Polman A (2008). Plasmonic solar cells. Opt Express.

[40] Pillai S, Green MA (2010). Plasmonics for photovoltaic applications. Sol Energy Mater Sol Cells.

[41] Jinfeng Z, Mei X, Ryan H, Faxian X, Baoqing Z, L WK (2012). Light concentration and redistribution in polymer solar cells by plasmonic nanoparticles. Nanoscale.

[42] Mie G (1908). Beiträge zur optik trüber medien, speziell kolloidaler metallösungen. Ann Phys.

[43] Tatsuya K, Ichiro F (1990). A treatment by the FD-TD method of the dispersive characteristics associated with electronic polarization. Microw Opt Technol Lett.

[44] Oubre C, Nordlander P (2004). Optical properties of metallodielectric nanostructures calculated using the finite difference time domain method. J Phys Chem B.

[45] Foteinopoulou S, Vigneron J, Vandenbem C (2007). Optical near-field excitations on plasmonic nanoparticle-based structures. Opt Exp.

[46] Kolwas K, Derkachova A, Demianiuk S (2006). The smallest free-electron sphere sustaining multipolar surface plasmon oscillation. Comput Mater Sci.

[47] Kolwas K, Derkachova A (2010). Plasmonic abilities of gold and silver spherical nanoantennas in terms of size dependent multipolar resonance frequencies and plasmon damping rates. Opto-Electron Rev.

[48] Njoki PN, Lim I-IS, Mott D, Park H-Y, Khan B, Mishra S, Sujakumar R, Luo J, Zhong C-J (2007). Size correlation of optical and spectroscopic properties for gold nanoparticles. J Phys Chem C.

[49] Haiss W, Thanh NTK, Aveyard J, Fernig DG (2007). Determination of size and concentration of gold nanoparticles from uv-vis spectra. Anal Chem.

[50] Sönnichsen C (2001) Plasmons in metal nanostructures. Ph.D. thesis, Ludwig-Maximilians-Universität München

[51] Etchegoin PG, Ru ECL, Meyer M (2006) An analytic model for the optical properties of gold. J Chem Phys 125(164705) 10.1063/1.2360270 17092118

[52] Grady N, Halas N, Nordlander P (2004). Influence of dielectric function properties on the optical response of plasmon resonant metallic nanoparticles. Chem Phys Lett.

[53] Blaber MG, Arnold MD, Ford MJ (2009). Search for the ideal plasmonic nanoshell: The effects of surface scattering and alternatives to gold and silver. J Phys Chem C.

[54] Zeman EJ, Schatz GC (1987). An accurate electromagnetic theory study of surface enhancement factors for Ag, Au, Cu, Li, Na, AI, Ga, In, Zn, and Cd. J Phys Chem.

[55] Vial A, Grimault A-S, Macias D, Barchiesi D, de la Chapelle M (2005) Improved analytical fit of gold dispersion: application to the modeling of extinction spectra with a finite-difference time-domain method. Phys Rev B 71(085416)

[56] Laroche T, Girard C (2006) Near-field optical properties of single plasmonic nanowires. Appl Phys Lett 89(233119)

[57] Hao F, Nordlander P (2007) Efficient dielectric function for fdtd simulation of the optical properties of silver and gold nanoparticles. Chem Phys Lett:115–118

[58] Pyykko P, Desclaux JP (1979). Relativity and the periodic system of elements. Acc Chem Res.

[59] Beversluis M, Bouhelier A, Novotny L (2003). Continuum generation from single gold nanostructures through near-field mediated intraband transitions. Phys Rev B.

[60] Urban AS (2010) Optothermal Manipulation of Phospholipid Membranes with Gold Nanoparticles. Ph.D. thesis, Ludwig-Maximilians-Universitat München̈

[61] Ehrenreich H, Philipp HR (1962). Optical properties of Ag and Cu. Phy Rev.

[62] Myroshnychenko V, Rodríguez-Fernández J, Pastoriza-Santos I, Funston AM, Novo C, Mulvaney P, Liz-Marzán LM, Garcia de Abajo FJ (2008). Modelling the optical response of gold nanoparticles. Chem Soc Rev.

[63] Ashcroft NW, Mermin ND (1976). Solid State Physics.

[64] Hövel H, Fritz S, Hilger A, Kreibig U (1993). Width of cluster plasmon resonances: bulk dielectric functions and chemical interface damping. Phys Rev B.

[65] Link S, El-sayed MA (1999) Size and temperature dependence of the plasmon absorption of colloidal gold nanoparticles 103:4212–4217

[66] Novo C, Gomez D, Perez-Juste J, Zhang Z, Petrova H, Reismann M, Mulvaney P, Hartland GV (2006) Contributions from radiation damping and surface scattering to the linewidth of the longitudinal plasmon band of gold nanorods: a single particle study. Phys Chem Chem Phys 8:3540–3546 10.1039/b604856k 16871343

[67] Hu M, Novo C, Funston A, Wang H, Staleva H, Zou S, Mulvaney P, Xia Y, Hartland GV (2008). Dark-field microscopy studies of single metal nanoparticles: understanding the factors that influence the linewidth of the localized surface plasmon resonance. J Mater Chem.

[68] Charles DE, Gara M, Aherne D, Ledwith DM, Kelly JM, Blau WJ, Brennan-Fournet ME (2011). Scaling of surface plasmon resonances in triangular silver nanoplate sols for enhanced refractive index sensing. Plasmonics.

[69] Berciaud S, Cognet L, Tamarat P, Lounis B (2005). Observation of intrinsic size effects in the optical response of individual gold nanoparticles. Nano Lett.

[70] Born M, Wolf E (1999) Principles of Optics. Cambridge University Press

[71] Khlebtsov NG, Dykman LA (2010) Optical properties and biomedical applications of plasmonic nanoparticles 111:1–35

